# Editorial: Neuromuscular Performance During Lifespan: Assessment Methods and Exercise Interventions

**DOI:** 10.3389/fphys.2019.01348

**Published:** 2019-10-24

**Authors:** Oliver Faude, Lars Donath

**Affiliations:** ^1^Department of Sport, Exercise and Health, University of Basel, Basel, Switzerland; ^2^Department of Intervention Research in Exercise Training, German Sport University Cologne, Cologne, Germany

**Keywords:** sensorimotor, strength, balance, postural control, gait, power, motor co-ordination

Neuromuscular performance can be regarded as the ability of the neuromuscular system to functionally control and drive movements by an appropriate integration, coordination and use of sensory feedback, reflex activity, central motor drive, muscle recruitment pattern, muscular excitation-contraction coupling, and energy availability (Faude et al., 2017). Proper neuromuscular performance enables the human organism to maintain stability and posture within the gravitational field in static and dynamic situations, to generate an appropriate amount of force for a given motor task or to co-ordinate limb movements to protect body structures and to avoid tissue damage, respectively. This definition can be used as a widely applicable umbrella term that subsumes a variety of different dimensions of physical function that determines the way to assess and train neuromuscular performance in different populations and settings.

It is accepted within the scientific community that a well-developed capacity of the neuromuscular system is highly relevant for fitness, sports, and health during the whole lifespan. In early years, the appropriate development of the neuromuscular capacity supports the acquisition of basic movement and motor skills and, thus, contributes to sports competency (Logan et al., 2012). Proper neuromuscular performance development may lead children and adolescents into an active and healthy lifestyle. Furthermore, the capacity of the neuromuscular system is fundamental to achieve peak sports performance in late adolescence and young adulthood (Granacher et al., 2016). In this regard, there is also convincing evidence that injury risk can be reduced by exercise interventions targeting particularly the neuromuscular system (Rössler et al., 2014). During later stages of life, a well-trained neuromuscular capacity enables people to stay active and healthy as well as maintaining the ability to fulfill the job requirements (Jakobsen et al., 2015). In elderly people, neuromuscular fitness can minimize the risk and rates of falling up to 50% (Sherrington et al., 2017). During the later stages of life, the capacity of the neuromuscular system remains relevant to deal with the demands of activities of daily life (ADL) and, thus, to stay mobile and independent as long as possible (McPhee et al., 2016).

Although the relevance of neuromuscular performance is widely recognized, there is a large diversity in assessment methods, cross-sectional associations between relevant outcome measures and potentially efficacious exercise interventions. Whereas, the aerobic capacity or muscular strength are very similarly assessed from childhood to older age, for instance, by conducting a VO_2max_ or one-repetition maximum test, respectively, there is no such uniform assessment method for neuromuscular performance measures. The diversity of assessment methods is mirrored by a large heterogeneity of intervention approaches. This phenomenon might be attributed to an anticipated task-specificity of neuromuscular co-ordination and adaptations with a lack of far transfer effects (Kümmel et al., 2016). A valid comparison of neuromuscular performance during different stages of the lifespan is, therefore, challenging and remains difficult.

Against the aforementioned background, the main aim of our Research Topic entitled “Neuromuscular performance during lifespan: assessment methods and exercise interventions” was to compile original research articles, systematic reviews and meta-analyses on topics related to neuromuscular performance during lifespan from a cross-sectional and longitudinal perspective. The scientific articles cover “neuromuscular performance” as a broad concept referring to different assessment methods and exercise interventions targeting neuromuscular performance in mainly healthy populations of different ages during lifespan.

In total, 19 scientific articles from 109 authors were finally accepted for publication (for an overview see Table 1). Of those, one article is an opinion paper, one a meta-analysis, one an open data set, and the remaining papers report original data. Thereof, 12 articles referred to data from cross-sectional studies and four studies applied longitudinal exercise training interventions. Seven articles referred to the neuromuscular domain of strength, strength-endurance, or muscular power, six dealt with gait or running analysis, four with balance and posture, and one with a general motor control topic and a methodological issue, each.

**Table 1:** Overview of published research papers categorized by different domains of neuromuscular performance, population, sample size, and study design.

**Domain**	**Population**	***N* (Age)**	**Design**	**Authors**
**STRENGTH, FORCE, AND POWER**				
Maximal eccentric hamstring strength	Youth/elite skier	170 (14–22 years)	X-sectional	Franchi et al.
Mechanical power output and jumping	Adults	55 (18–67 years)	X-sectional	König et al.
WB-EMS (leg power, jumps, and sprints)	Young adults	19 (25 years)	Intervention (8 weeks)	Micke et al.
Transversal calf muscle loading	Young adults	15 (26 years)	X-sectional	Siebert et al.
Neuromuscular function and dehydration	Youth combat athletes	14 (25 years)	X-Sectional	Barley et al.
Force-velocity and flexibility	Female runner	33 (40 years)	X-sectional	Nikolaidis et al.
Changes of maximal leg strength	Adults to seniors	362 (19–91 years)	X-sectional	Kemmler et al.
**GAIT, RUNNING, STEPPING, AND AGILITY**				
Modular control of running	Adults	135 (30 years)	Open Data Set	Santuz et al.
Walking based agility testing	Seniors	66 (69 years)	X-sectional	Lichtenstein et al.
Stepping down motor strategies	Seniors	21 (71 years)	X-sectional	Kluft et al.
Sensor based attractor analyses	Clinical seniors	43 (72 years)	X-sectional	Byrnes et al.
Factors affecting gait stability	Seniors	102 (72 years)	X-sectional	Hamacher et al.
Systems theory and locomotion	–	–	Opinion Article	Van Hooren et al.
**BALANCE AND POSTURE**				
Correlations between balance tasks	Children to seniors	9,353 (6–93 years)	Meta-analysis	Kiss et al.
Reactive balance training	Adults	39 (24 years)	Intervention (4 weeks)	Krause et al.
Interference and balance task learning	Adults	69 (25 years)	Intervention (2 weeks)	Giboin et al.
Athletic training and body perception	Adolescents	67 (14–20 years)	Intervention (6 years)	Ludwig et al.
**OTHERS**				
Force output and network dynamics	Older adults	47 (55 years)	X-sectional	Gölz et al.
Intermuscular coherence/EMG	Adults	18 (26 years)	X-sectional	Mohr et al.

*WB-EMS, whole-body electromyostimulation*.

In the strength and power domain, Kemmler et al. report cross-sectional data of maximum isokinetic knee extension and flexion strength in 362 non-athletic male adults within an age range from 19 to 91 years. The authors found a small decrease in maximum isokinetic leg strength of 0.15% (knee extension) to 0.5% (knee flexion) per year up to the age of 50–60 years and, thereafter an accelerated annual loss of about 1.3%. These results can be used as normative values for healthy male populations of different age groups and point toward the need for sarcopenia prophylaxis in men beginning in the 5th decade in order to address the accelerated muscle decline of advanced age. Nikolaidis et al. and Franchi et al. present normative data for specific athletic populations. Franchi et al. show maximal eccentric hamstring strength data of 170 competitive alpine skiers from the under-15 age group up to elite level skiers. The authors arrive at the conclusion that these data emphasize the relevance of considering maturation when interpreting eccentric hamstring strength data and that such data may support injury prevention approaches. Nikolaidis et al. analyzed force-velocity characteristics, muscle strength, and flexibility in female recreational marathon runners and their relationship with age, race time, and anthropometric characteristics. Although anaerobic power and neuromuscular fitness of female marathon runners are not directly linked to race performance, these findings may be useful for strength and conditioning coaches to monitor the training of athletes, particularly from a more general health-related physical fitness perspective. With regard to potential mechanisms for age-related changes in muscular power, König et al. examined triceps surae muscle strength and tendon stiffness in middle-aged (40–67 years old) compared to younger (18–30 years of age) healthy male adults. In a strength- and stiffness-matched sub-group, the authors analyzed drop jump performance. The authors conclude that “the reduced muscular power output during lower limb multi-joint tasks seen with aging may be due to age-related changes in motor task execution strategy rather than due to muscle weakness.”

Acute intervention effects on strength-related outcomes were studied by Barley et al. as well as Siebert et al.. Barley et al. reported that acute dehydration by 3.2% of body mass resulted in impaired muscle strength-endurance and increased fatigue perception in combat sports athletes. Markers of central and peripheral functioning were, however, not altered. Siebert et al. analyzed effects of multidimensional transversal loads around the calf on isometric force during plantar flexions. The results indicate that proprioception may be enhanced and muscle oscillations reduced, which may lead to improved performance. Whether compression garments may impact more complex sports performance remains elusive to date and could be interesting for further research.

Micke et al. conducted a randomized controlled trial on the effects of superimposed whole-body electromyostimulation training vs. traditional strength training on strength and power parameters in male sport students. The results indicate that the combination of dynamic exercises with whole-body electromyostimulation can be as effective as dynamic resistance training alone. A specific “add-on” effect of whole-body EMS cannot be generally assumed.

Van Hooren et al. submitted a highly interesting opinion paper (most viewed paper of this Research Topic as per June 2019) in which they propose a theoretical framework applying dynamical systems theory in order to improve human locomotion. They aimed at, for instance, maintaining gait stability or reducing injury risk. Human locomotion, thereby, can be regarded as a self-organized process based on various attractors on a macroscopic (walking, running), mesoscopic (joint coupling), and microscopic (neural activity and central pattern generators) level, respectively. This framework can serve as a theoretical model, which can be used for studying risk factor models for falls in seniors, for injury prevention in sports or the effectiveness of exercise interventions. Hamacher et al. studied the effects of a number of potentially influencing factors [health and pain status, fear of falling, depression, cognition performance, physical activity, proprioception (joint position sense), peripheral sensation, balance performance, and muscular fitness] on gait stability in 102 adults older than 65 years of age. The authors concluded that the ability to recover from small perturbations may be related to physical activity, peripheral sensation, and pain status. Kluft et al. observed that older adults do not necessarily select a certain motor strategy, which is associated with their physical abilities. They rather have an imprecise perception of their physical abilities during unexpected stepping down movements. It is of crucial interest to further evaluate whether this inappropriate selection of a motor strategy can explain accidental falls in older adults. In this line of reasoning, we previously proposed an agility-based approach to study fall risk and prevention in older adults (Donath et al., 2016). In this regard, Lichtenstein et al. evaluated the reliability and validity of a newly developed test parcour (ACE, Agility Challenge for the Elderly). The walking-based ACE test and its sub-domains (stop and go, cutting, spatial orientation) differentially reflect cardiocirculatory and neuromuscular domains with cardiocirculatory fitness and gait speed contributing most to overall performance. The test, thus, can be useful for documenting changes due to exercise interventions in seniors.

Santuz et al. provided an open-access data set (available at Zenodo; doi: 10.5281/zenodo.1254380) with EMG activities recorded during treadmill running in 135 healthy young adults of both genders. According to the authors, this data set (i) can be used as a prime source for expanding the representation of human motor control due to the large data set, (ii) it can support scientists from multiple disciplines (e.g., musculoskeletal modeling, robotics, neuroscience, sport science), and (iii) it can be used to train students and scientists with muscle synergy extraction methods.

Byrnes et al. analyzed the attractor variability and pattern for acceleration gait data in healthy adults compared to patients with symptomatic lumbar spinal stenosis. There was a difference in attractor patterns between healthy people and patients, but also a large variation in the attractor gait data within groups. Although the attractor used in this study reflects pathology, the variability is too large for a reliable application in this patient population.

The most viewed original article (June 2019) is a randomized controlled trial on the efficacy of reactive balance training as compared to conventional balance training on neuromuscular and kinematic parameters which are relevant with regard to fall risk. Krause et al. found enhanced reflex activity in the leg muscles and improved neuromuscular timing and accuracy in both groups, potentially resulting in more efficient segmental stabilization during fall risk situations. In perturbed situations, the effects were more pronounced and effect sizes larger in the group which performed reactive balance training, pointing toward a high specificity of balance training adaptations. Similarly, the results of Giboin et al. suggest that balance training adaptations are specific and transfer effects are unlikely. Within two different experiments, the authors observed that neither additional intra- nor intersession tasks interfere with the learning of a novel balance task. A meta-analysis of Kiss et al. compared the correlations between different types of balance performance (static, dynamic, proactive, and reactive) in healthy populations during lifespan. The authors observed merely small correlations between any type of balance performance, again indicating that balance is rather task-specific than a “general” ability. This result underpin the need of multiple tests or exercises for the assessment and training-induced changes of balance performance.

Ludwig et al. reported longitudinal data on the development of body posture over a 6-year period in 67 adolescents from age 14 to 20 years. A sub-group performed a specific posture training twice per week in addition to all other physical and sports activities. The results of this long-term longitudinal study suggest that “additional athletic training of 2 h per week including elements for improved body perception seems to have the potential to improve body posture in symptom free male adolescents and young adults.”

Although it is well-known that continuous deliberate practice throughout lifespan leading to motor expertise can delay age-related deteriorations in motor skills, relatively little is known about underlying mechanisms. Whether acquired motor expertise leads to a higher initial status from which the age-related decline starts or whether motor expertise results in qualitative differences in motor output and neural processing was the main research question of Gölz et al. A relevant topic from a healthy aging perspective. The authors studied the precision of fine motor control in experts compared to novices with regard to the execution of a dynamic force control task. The obtained results suggest that motor performance of experts is more precise, less variable, and more complex. This finding points toward a better performance and adapted organization of sensorimotor control.

Electromyographical (EMG) assessment of muscle activity can give important insight into the co-ordination and synchronization of muscles during human movements and, consequently, may enable an understanding of muscle performance. Mohr et al. evaluated whether the choice of electrode configuration (monopolar vs. bipolar) or amplifier technology (potential vs. current) have a relevant effect on intermuscular EMG coherence between two muscles (mm. vastus lateralis and medialis) during stable and unstable squatting. The authors indeed found large methodological differences that can explain inconsistencies, which were observed in the scientific literature and should be considered in future studies on muscle activity.

Beside aerobic capacity, a well-developed neuromuscular capacity can be considered a crucial motor performance domain across all age-groups at different performance levels and settings. Different entities refer to “neuromuscular capacity” ranging from strength, power, balance and gait to stepping, running, and agility performance. Numerous behavioral (e.g., postural sway, strength, power, jumping height) and mechanistical (e.g., EEG, EMG) methodological approaches have been developed to “parameterize” neuromuscular performance and capacity, respectively. Standard tools to measure those entities in a valid and reliable manner are highly required and need to be developed and justified based on the intended goals and intentions. The majority of the published papers of this Research Topic are of cross-sectional nature underpinning that walking, stepping and gait are specific motor tasks that involve different strategies on spinal and supra-spinal level. To explain the large variability and specificity of these complex motor tasks theoretical frameworks and one data set have been published. Established behavioral surrogates have been linked with physical and sports performance as well as fall risk and were investigated in both cross-sectional and longitudinal study designs: thereby, for example, the association between performance and dehydration, eccentric strength and maturation, athletic training and body perception, mechanical power and jumping performance, strength and transversal calf muscle load, force velocity and flexibility as well as agility, and neuromuscular performance have been found to be interesting to investigate more detailed. Few studies investigated neuromuscular interventions. Unfortunately, most of these studies lasted <8 weeks. Although interventional studies are methodologically challenging, more studies over longer period of time (e.g., 1 year) are mandatorily needed. This is particularly important as we justify task-specific neuromuscular adaptations mostly based on short time frames of around 12 weeks or less. However, based on more than 9,000 included subjects within the meta-analyses and more than 1,000 investigated subjects within the original papers, the relevance of neuromuscular performance investigated from different perspectives using different approaches across all age groups can be considered very high. The findings of this Research Topic emphasize that neuromuscular motor performance tasks from upright standing to downhill skiing are complex and specific tasks that reflect different neuromuscular requirements. Keeping the different measured or targeted entities of “neuromuscular performance” in mind, it appears challenging to derive gold standard tests to assess neuromuscular performance in general, based on the available papers of this Research Topic. It remains still difficult to compare several tools used in different papers to assess neuromuscular performance as generalizable physical capacity. Future research still needs to rationalize and conceptualize, which outcome needs to be tested and trained in the light of the population, setting, and intended goals. This information should than have impact on interventional study designs that could also focus on primary (hard) endpoints such as fall, disease, mortality, performance, maturation, and aging. These findings should then be incorporated into modeling frameworks that could provide a more holistic and integrative understanding of complex neuromuscular motor tasks.

## Author Contributions

All authors listed have made a substantial, direct and intellectual contribution to the work, and approved it for publication.

### Conflict of Interest

The authors declare that the research was conducted in the absence of any commercial or financial relationships that could be construed as a potential conflict of interest.
